# Epigenetic Regulation of Sox30 Is Associated with Testis Development in Mice

**DOI:** 10.1371/journal.pone.0097203

**Published:** 2014-05-08

**Authors:** Fei Han, Yan Dong, Wenbin Liu, Xuexiang Ma, Ronghui Shi, Hongqiang Chen, Zhihong Cui, Lin Ao, Huidong Zhang, Jia Cao, Jinyi Liu

**Affiliations:** Institute of Toxicology, College of Preventive Medicine, Third Military Medical University; Key Laboratory of Medical Protection for Electromagnetic Radiation, Ministry of Education of China, Chongqing, PR China; Clermont Université, France

## Abstract

DNA methylation is involved in tissue-specific and developmentally regulated gene expression. Here, we screened a novel methylation gene *Sox30*, whose methylation might contribute to its regulation and testis development in mice. *Sox30* is a member of Sox transcription factors, and is considered to be involved in spermatogonial differentiation and spermatogenesis. However, the precise function and regulatory expression pattern remain unclear. In the present study, we found that *Sox30* is highly expressed in adult testes but not in ovaries. Sox30 expression begins in early development, and in the testes, it is specifically increased coincidentally with development until adulthood. Moreover, Sox30 is expressed not only in testis germ cells, but also in sertoli cells. *Sox30* is hypo-methylated in testis, epididymis and lung of adult mice, in which *Sox30* is expressed. By contrast, *Sox30* is hypermethylated in ovary, heart, brain, liver, kidney, spleen, pancreas, muscle, intestine, pituitary gland, blood and hippocampus of adult mice, in which the *Sox30* is absent. Importantly, decreased methylation at CpG islands of *Sox30* is observed in mouse developmental testes after birth, which is associated with enhanced Sox30 expression. However, the hypermethylated status of Sox30 is maintained in ovaries that does not express Sox30 during this period. Further, following demethylation treatment using 5-aza-dC, *Sox30* expression is restored in GC2, TM3 and TM4 cell lines. This observation convincingly confirms that methylation really contributes to *Sox30* silencing. In summary, we show that Sox30 expression is under the control of DNA methylation status, and this expression pattern is associated with testis development in mice.

## Introduction

Epigenetic systems underlie the transcription factor networks, which establish gene expression in response to tissues and developmental stages [1]. In these systems, DNA methylation is one of the most major common players [2]. In mammals, formation of DNA methylation pattern is one of the important epigenetic events and is essential for development of various tissues [3]. Previous studies have revealed that DNA methylation pattern is specific to cell type, and is associated with cell differentiation [4], [5]. DNA methylation is always involved in various gene regulatory processes by silencing, switching and stabilizing genes, as well as remodeling chromatin [6]–[14].

The Sox family of transcription factors encode proteins that are characterized by a sequence-specific DNA binding HMG-box, and are a group of genes related to the mammalian testis determining factor, Sry. In general, the proteins containing an HMG domain with more than 50% similarity in the HMG domain of Sry are considered as Sox members. At present, about 20 different Sox genes have been identified in mammalian [15]. Accumulating evidence showed that Sox members play key roles in a wide variety of developmental processes, including sex determination and differentiation, testis development, male fertility maintenance and other respects [16], [17].

*Sox30*, a member of the Sox family, was firstly cloned from mouse (*Mus musculus, location Chr 11*) and human (*Homo sapiens, location Chr 5*), and then form Nile tilapia [18], [19]. In mouse and human, *Sox30* is considered to be involved in spermatogonial differentiation and spermatogenesis [18], [20]. In the Nile tilapia, *Sox30* plays roles in gonadal development [19]. However, up to now, the precise function and regulatory expression pattern of *Sox30* have remained unknown.

In the present study, we first revealed that Sox30 was expressed both in mouse testis germ cells and sertoli cells, and the CpGs of *Sox30* were specifically demethylated coincident with the spatiotemporal pattern expression found in mouse testis development. Further, re-expression of silenced *Sox30* was found after treatment of cell lines with 5-aza-dc, a pharmacological inhibitor of DNA methylation, substantiating the role of methylation in the silencing of *Sox30*. The data demonstrate that DNA methylation plays key role in transcriptional regulation of Sox30 in testis development of mice.

## Materials and Methods

### Cell lines

The GC2 (Spermatocyte, GC-2spd), TM3 (Leydig cell) and TM4 (Sertoli cell) cell lines were obtained from the American Type Culture Collection (ATCC, Manassas, VA, USA), cultured in DMEM or Ham's F12/DMEM (HyClone) supplemented with 10% fetal bovine serum or 5% horse serum/2.5% fetal bovine serum (FBS) (HyClone), and incubated in an atmosphere of 5% CO_2_ in air at 37°C.

### Animals

BALB/c mice of both sexes were obtained from the animal center of Daping Hospital, Third Military Medical University (Chongqing, China). Mice were housed in cages, at a constant temperature (25°C), with a relative humidity of 50–60%, and a 12 h dark/light cycle. Food and water were given *ad libitum*. All experiments conformed to the Guide for the Care and Use of Laboratory Animals and were approved by the Institutional Committee of Laboratory Animal Experimentation at Third Military Medical University, China.

### Sertoli and germ cells separation and purification

Sertoli cells were separated from 18 days old male mouse testes according to previous procedure [21]–[24]. Trypsin (Hyclone) and collagenase IV (Sigma) were used to digest the testis tissue for 30 min at 37°C separately. Dulbecco's Modified Eagle's Medium/Ham's Nutrient Mixture F12 (DMEM/F12, 1:1) medium (Hyclone) containing 10% FBS (Hyclone) was used to finish the digestion. The enzyme-digested product was sieved under 80 meshes. Primary separated cells were cultured in 6 centimeters diameter dishes (Corning) with DMEM/F12, 10% FBS and 1× Penicillin-Streptomycin (Beyotime, China) in a humidified atmosphere of 5% CO_2_ at 35°C. Germ cells (medium) were collected to another dish for culture [25]–[29]. Then adherent cells were treated with 20 mM Tris-HCl (pH 7.4) for 2 minutes after 48 hr of culture.

### Genomic DNA extraction

DNA was extracted from the various tissues and cell lines with a Promega DNA Purification Wizard kit (Promega) according to the manufacturer's instructions. The samples were collected into lysis buffer with proteinase K, digested at 55°C overnight, and then incubated at 95°C for 10 min. The DNA was then precipitated with ethanol, resuspended in 50 µl deionized water and stored at −20°C until analysis.

### Total RNA extraction

Total RNA was isolated from various tissues (mixed tissues from five individuals) and cell lines using Trizol reagent (Invitrogen) according to the manufacturer's instructions. The RNA (2.0 µg) was treated with DNase I to eliminatethe genomic DNA contamination. Then cDNAs were synthesized using M-MLV First Strand Kit (Invitrogen), and stored at −20°C.

### Analysis of *Sox30* expression by reverse transcription-polymerase chain reaction (RT-PCR)

RT-PCR was performed according to methods described previously [30]. The templates were set with Sox30 plasmid DNA and deionized water for positive and negative controls, respectively. The β-actin was amplified as internal control. A series of PCRs with different cycles (from 18 to 36, with an interval of 2) were performed to determine the linear phase using 1 µl cDNA. Based on these pilot experiments, 20 cycles for β-actin and 32 cycles for Sox30 were chosen. The primers used for RT-PCR are listed in Table 1.

**Table 1:** Sequence of primers used in the present study.

Primer	Sequence	Purpose
M-Sox30F	GCCTCGTCCAGGGAAGCGAA	Expression analysis (RT-PCR)
M-Sox30R	CCGAAGGGAGCCTATGAATGTC	
MSox30F(M)	GGAGAGAGTTAGGTCGGAGTTATC	MSP analysis
MSox30R(M)	CGACTTCACTTACAACAAACGTC	
MSox30F(U)	TGGAGAGAGTTAGGTTGGAGTTATT	
MSox30R(U)	TTCCAACTTCACTTACAACAAACAT	
MSox30F(B)	TTTTTTTTATGGAGAGAGTTAGGT	BSP analysis
MSox30R(B)	AAAACAACAACAACACCTATTCC	
Sox30F	CCCATTCCACACTCACACGTCTA	Expression analysis (Real Time PCR)
Sox30R	AACCAAGACATTCTGGCATTGAACT	
β-actin-F	GGAGATTACTGCTCTGGCTCCTA	Internal control
β-actin-R	GACTCATCGTACTCCTGCTTGCTG	

### Analysis of *Sox30* expression by RT- quantitative PCR (RT-qPCR)

The cDNAs were prepared specific for RT-qPCR using PrimeScript RT reagent Kit With gDNA Eraser (Perfect Real Time) (Takara, RR047A). Real-time PCR was carried out using an iQ5 real-time detection system (Bio-Rad Laboratories) and SYBR Premix Ex TaqII (Perfect Real Time) (Takara, RR820A) according to manufacturer's instructions. The mRNA levels were normalized to β-actin and the 2^−ΔΔt^ method was used to analyze the relative levels of expression. The primers used for RT-qPCR are listed in Table 1.

### Western blotting (WB) analysis

Protein (80 micrograms) extracted from samples was run on 10% SDS–PAGE and transferred to PVDF membrane (Millipore). The membrane was blocked for 2 hr at room temperature, and incubated overnight at 4°C with primary antibody (Sox30; 1∶1000; Santa Cruz Biotechnology, sc-20104). The membranes were then washed, incubated with secondary antibody (1∶4000, Jackson ImmunoResearch Laboratories) and developed with SuperSignal West Pico Chemiluminescent substrate (Pierce). The same membrane was stripped and incubated with β-actin antibody (Sigma), serving as an internal control.

### Immunohistochemistry (IHC) analysis

The paraffin-embedded tissue sections were baked at 60°C for 2 h, deparaffinized in xylene, rehydrated through graded alcohol to water, immersed in citrate buffer (pH 6.0), and antigen retrieval accomplished at 95°C for 15 min. The sections were blocked with 0.3% H_2_O_2_, treated with 10% normal goat serum for 15 min, and incubated at 4°C citrate buffer overnight with Sox30 antibody (1∶50; Santa Cruz Biotechnology). The reaction of antigen–antibody was visualized using streptavidin-horseradish peroxidase conjugated with diaminobenzidine after reaction with a biotinylated secondary antibody. The section incubated with PBS instead of primary antibody was used as a negative control.

### Methylation analysis of *Sox30* by methylation-specific polymerase chain reaction (MSP) and bisulfite sequencing PCR (BSP) sequencing

DNA samples were modified using EZ DNA Methylation-Gold Kit (Zymo Research, D5005). The MSP, BSP and sequencing were performed as previously reported [31]. Two pairs of MSP primers were designed (one pair in the promoter region and other pair in Exon 1 region), but only one pair of MSP primers in Exon 1 region was amplified successfully. Primers for MSP and BSP are listed in Table 1.

### Treatment of cell lines with 5-aza-2-deoxycytidine (5-aza-dC)

To assess whether the expression of Sox30 was restored by 5-aza-dC treatment, demethylation experiments were performed. Briefly, GC2, TM3 and TM4 cells with Sox30 hypermethylation were exposed to 5-aza-dC (10 µM, Sigma) for three days, with media and drugs being replaced daily. The controls were treated in parallel with DMSO at each time point. DNA and total RNA were extracted, and the methylation status and mRNA expression of Sox30 were detected. The primer sequences used are shown in Table 1.

### Statistical analysis

Statistical analyses were performed with the SPSS version 16.0 software. The results were expressed as the mean ± standard error (S.D). The differences between groups were analyzed by the Chi-square test and the Student's t-test. All p values are 2-sided, and p values less than 0.05 were taken as statistically significant.

## Results

### Sox30 was highly expressed in testes of adult mice

**Figure 1:**
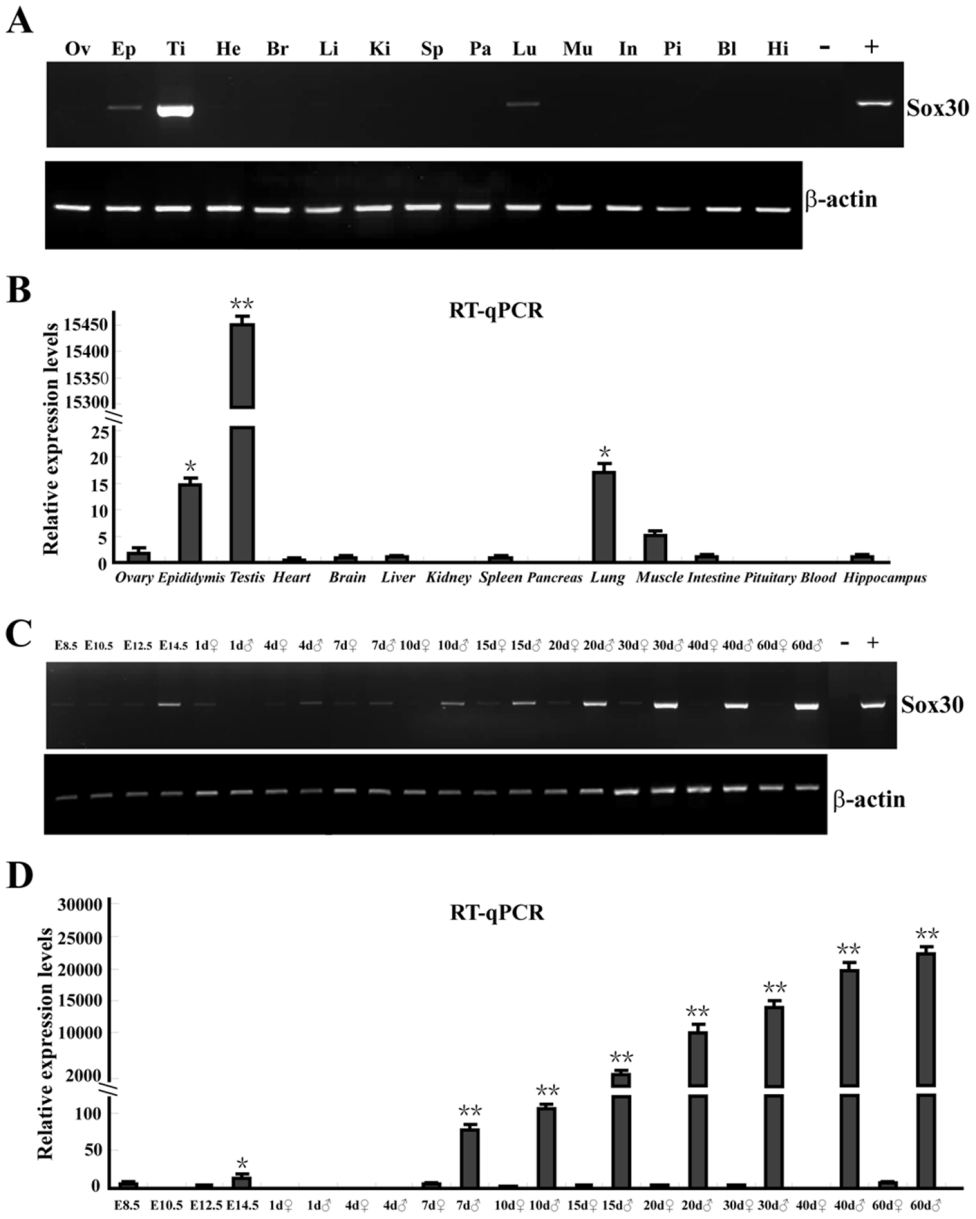
Expression patterns of *Sox30* in various adult tissues and developmental stages of the gonads. (A) Expression analysis of *Sox30* from various tissues of adults by RT-PCR. Ov, ovary; Ep, epididymis; Ti, testis; He, heart; Br, brain; Li, liver; Ki, kidney; Sp, spleen; Pa, pancreas; Lu, lung; Mu, muscle; In, intestine; Pi, pituitary; Bl, blood; Hi, hippocampus; −, negative control (deionized water); +, positive control (Sox30 plasmid DNA in place of the template); β-actin was used as the internal control. (B) Expression of *Sox30* in various tissues of adults by RT-qPCR. Error bars indicate standard deviation (S.D). (n = 3); * <0.05; **<0.01. (C) Ontogeny of *Sox30* expression in mouse gonads by RT-PCR. E8.5, embryonic day 8.5; 1d, 1 day post partum; ♀, female; ♂, male. (D) Ontogeny of Sox30 expression in mouse gonads by RT-qPCR. Error bars indicate S.D. (n = 3); * <0.05; **<0.01. Each sample of adult tissue and developmental stage gonad is a mix tissue from five individuals.

Tissue distribution analysis using RT-PCR and RT-qPCR revealed that *Sox30* was expressed in adult testis, epididymis and lung, but was absent from ovary, heart, brain, liver, kidney, spleen, pancreas, muscle, intestine, pituitary, blood and hippocampus. The expression of *Sox30* in testes was much higher (about 1000-fold) than that in epididymis and lung (Figure 1A, 1B).

### Sox30 was specifically increased coincidentally with testis development

**Figure 2:**
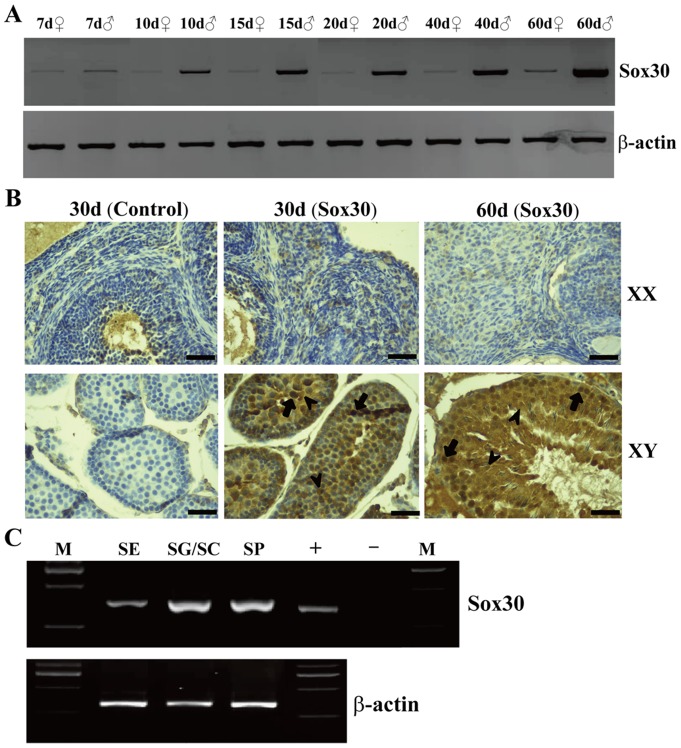
Expression of Sox30 in developmental gonads and cell types of testes. (A) Ontogeny of Sox30 protein expression in mouse testes and ovaries (mix tissues from five individuals) by WB. 7d, 7 day post partum; ♀, female; ♂, male; β-actin was used as the internal control. (B) IHC analysis of Sox30 expression in gonads at 30d and 60d. XX, female; XY, male; Control, negative control (PBS in place of a primary antibody); Arrow, signals of Sox30; Scale bars are 50 µm. (C) Sox30 expression in germ cells and sertoli cells of male gonads by RT-PCR; SE, sertoli cells; SG, spermatogonia; PS, primary spermatocyte; SP, spermatid; −, negative control; +, positive control; M, marker; β-actin was used as the internal control.

The expression of *Sox30* was detected in the whole embryos at E8.5 (embryonic day 8.5), E10.5, E12.5 and E14.5, and the gonads at 1d (1 day post partum), 4d, 7d, 10d, 15d, 20d, 30d, 40d and 60d of mice by RT-PCR, RT-qPCR and WB. *Sox30* expression was detected at low levels in the whole embryos at E8.5, E10.5, E12.5 and gonads at 1d, while at relatively high level in the whole embryos at E14.5 (Figure 1C, 1D). Thereafter, *Sox30* expression in the testes was specifically increased coincidentally with growing developmental stages until adult (4d, 7d, 10d, 15d, 20d, 30d, 40d and 60d), and a sharp increase of Sox30 expression was observed between 15d and 20d. However, the low expression status of Sox30 was maintained in ovaries during this period (Figure 1C, 1D and Figure 2A).

### Sox30 was expressed in germ cells and sertoli cells of mouse testes

To ascertain which population of cells expresses Sox30, IHC was performed using mouse gonads at 30d and 60d. Specific signals were observed in germ cells including spermatogonia and spermatocytes, and sertoli cells of the testes. However, no signal was detected in the ovaries (Figure 2B). To further confirm this result, germ cells and sertoli cells were separated and purified from 18 days old mouse testes. RT-PCR analysis showed that *Sox30* was indeed expressed in germ cells (high level) and sertoli cells (lower level) of the testes in mice (Figure 2C).

### The tissue-specific expression of Sox30 was associated with its methylation in adult mice

**Figure 3:**
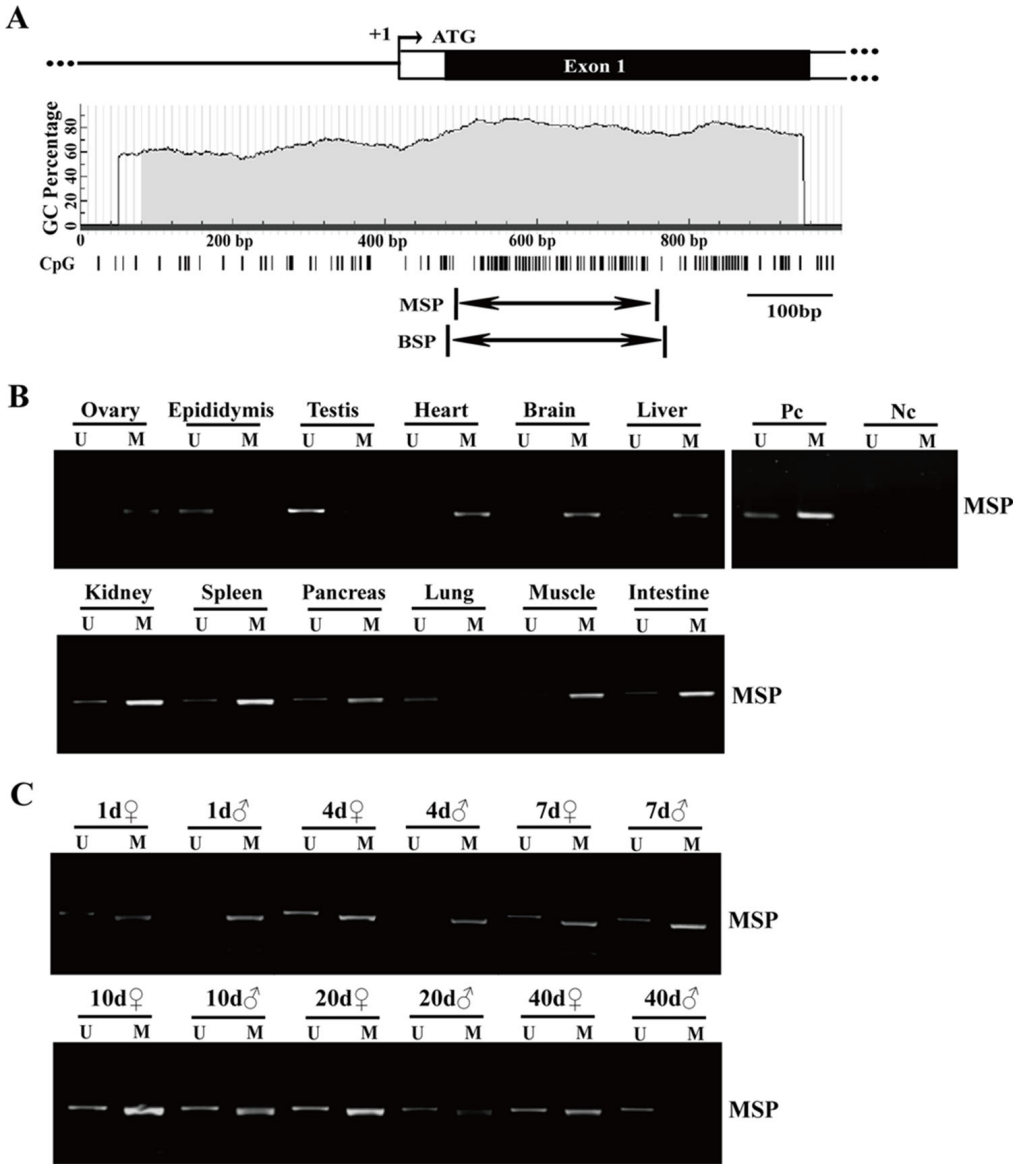
Methylation status of *Sox30* in various tissues and developmental gonads. (A) Schematic representation of the mouse *Sox30*. Open boxes, non-coding regions; Closed boxes, coding regions; Arrow, the transcriptional start site (+1); ATG, start codon; Vertical bars, CpG sites; Arrows below the CpG sites, the regions subjected to MSP and BSP. (B) The methylation status was analyzed by MSP in various tissues of adult mice. U, unmethylated-specific primers; M, methylation-specific primers; Pc, positive control, including fully unmethylated control (blood genomic DNA sample) and fully methylated control (blood genomic DNA sample treated with SssI-methylase); Nc, negative control (deionized water). (C) The methylation analysis by MSP was performed in developmental gonads of mice. U, unmethylated-specific primers; M, methylation-specific primers; ♀, female; ♂, male.

To determine whether tissue-specific expression of *Sox30* was correlated with its methylation, the methylation patterns of *Sox30* were detected. Pairs of MSP and BSP primers were showed in Figure 3A. The MSP analysis showed that hyper-methylation of *Sox30* was detected in ovary, heart, brain, liver, kidney, spleen, pancreas, muscle, intestine, pituitary, blood and hippocampus, but was absent from the testis, epididymis and lung (Figure 3B). Consequently, *Sox30* was silenced in the ovary, heart, brain, liver, kidney, spleen, pancreas, muscle, intestine, pituitary, blood and hippocampus, but was expressed in the testis, epididymis and lung (Figure 1A, 1B). These data suggested that *Sox30* expression was likely associated with its methylation state in various tissues of adult mice.

### The stage-specific expression of Sox30 was correlated with its methylation in developmental testes

To investigate whether the relationship between *Sox30* methylation and its expression also existed in mouse developmental gonads, the differential and developmental stage-specific methylation of *Sox30* was studied in gonads. Hypermethylation of *Sox30* was maintained in ovaries from 1d to 40d. By contrast, decreased methylation was observed in testes from 1d to 40d (Figure 3C). Taking the ontogeny results into consideration, *Sox30* expression was correlated to its methylation state in developmental gonads, and this decreased methylation in developmental testes was associated with enhanced expression of Sox30.

### De-methylation treatment with 5-aza-dC restored Sox30 expression

**Figure 4:**
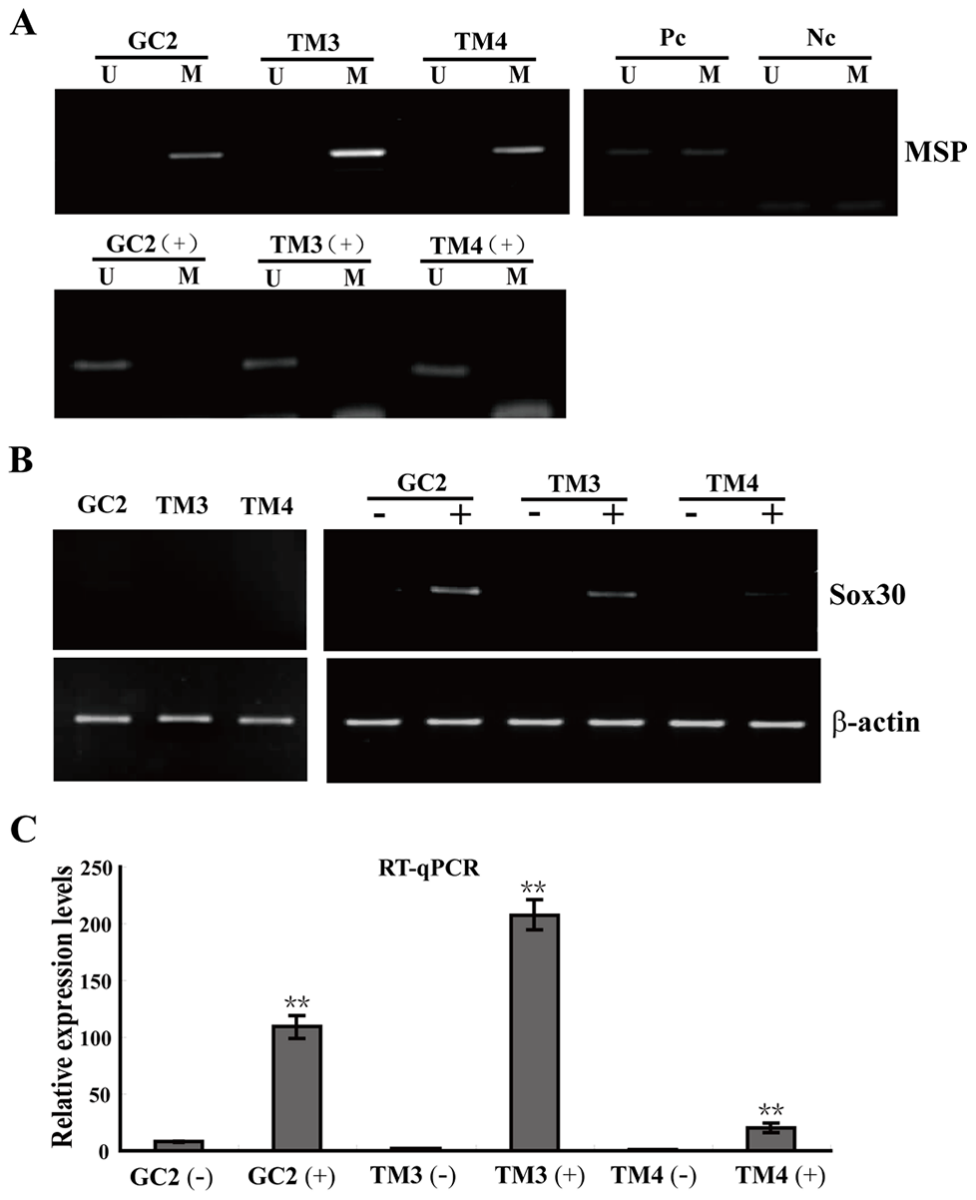
De-methylation analysis of *Sox30* in cell lines from gonad. (A) The electrophoresis results of MSP products from GC2, TM3 and TM4 cells. U, unmethylated-specific primers; M, methylation-specific primers; Pc, positive control; Nc, negative control; (+), Cells treated with 5-aza-dC (10 µM). (B) *Sox30* expression was analyzed in cell lines by RT-PCR. *Sox30* expression significantly increased after treatment with 5-aza-dC; (+), treated with 5-aza-dC; (−), treated with DMSO and without 5-aza-dC; β-actin was used as an internal control; (C) The expression of *Sox30* transcripts significantly increased after 5-aza-dC treatment as shown by RT-qPCR. Error bars indicate S.D. (n = 3).

To further confirm the methylation being associated with inhibition of *Sox30* expression, we detected *Sox30* mRNA level and methylation status in GC2, TM3 and TM4 cell lines that was incubated with/without 5-aza-dC (a DNA methylation inhibitor). Hypermethylation and decreased expression of *Sox30* were detected in the three cell lines (Figure 4A, 4B). After 5-aza-dC treatment, methylated alleles of Sox30 were not found in any of the cell lines (Figure 4A). As expected, the cell lines, which initially showed hypermethylation of *Sox30* and loss of its expression, were re-expressed *Sox30* after 5-aza-dC treatment (Figure 4B, 4C). These data strongly suggested that methylation of *Sox30* directly contributed to the regulation of *Sox30* expression.

### The MSP results were confirmed by BSP sequencing

**Figure 5:**
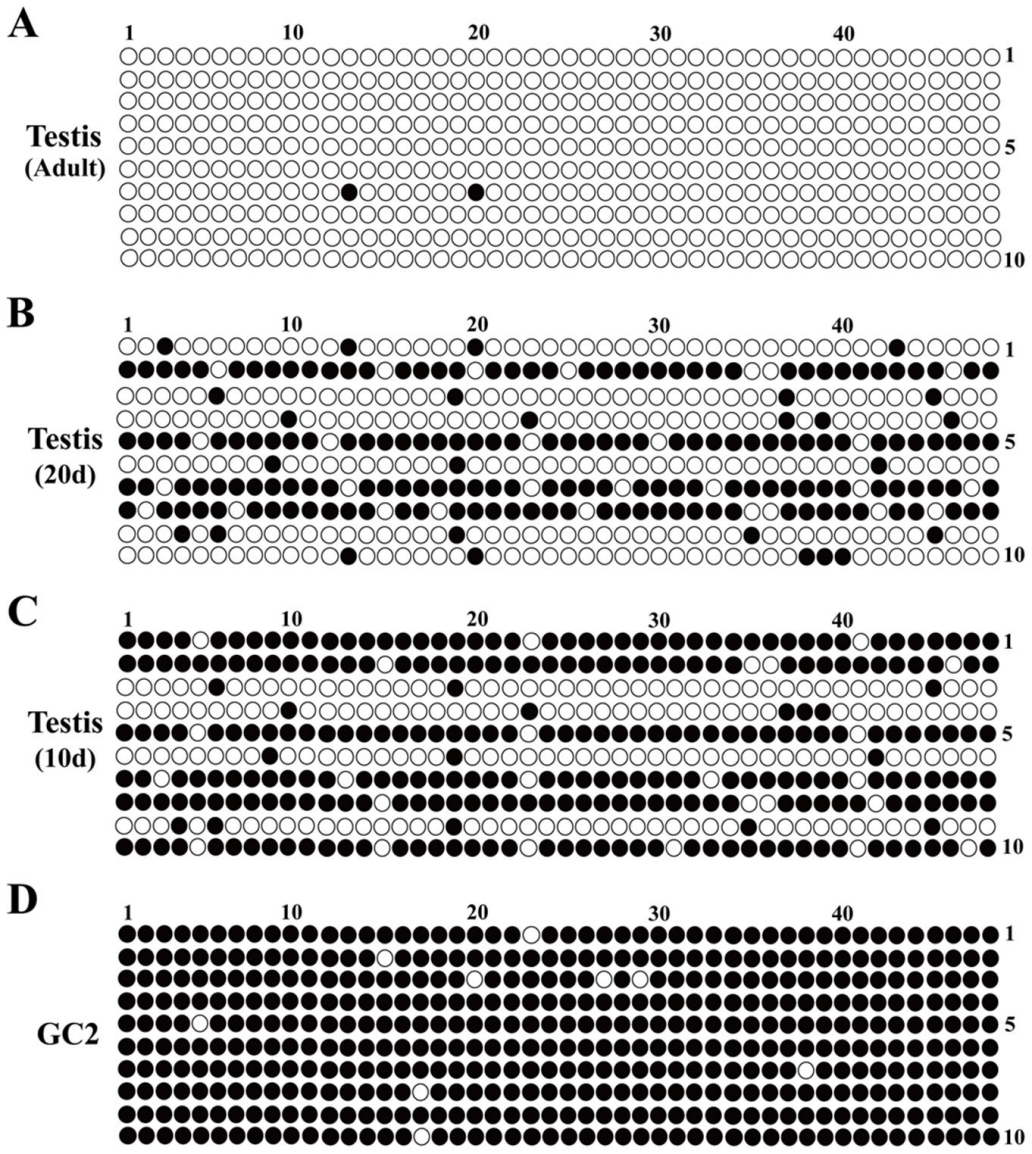
Methylation status of *Sox30* was confirmed by BSP sequencing. BSP sequencing of *Sox30* was performed to confirm MSP results in 60d old adult testis (A), 20d old testis (B), 10d old testis (C) tissues and GC2 cells (D). Number of rows, number of clones; Number of columns, number of CpG; Solid circles, methylated CpG sites; Open circles, unmethylated CpG sites.

In order to make the results more reliable, the MSP results above were further validated by BSP sequencing. Amplified fragments were cloned into a specific vector, and 10 clones were sequenced for each amplification product. The sequencing results were consistent with those of MSP, in which dense methylation was found in methylated GC2 cells and testis tissues at 10d and 20d but not in unmethylated adult testis tissues (Figure 5A–5D). Moreover, decreased methylation level of *Sox30* was observed in testes from 10d, 20d and adult mouse (60d) (Figure 5A–5C).

## Discussion

Tissue distribution analysis showed that *Sox30* was expressed in the testis, epididymis and lung of adult mice, and was highly expressed only in the testes. Moreover, the expression levels of Sox30 in the testes were nearly 1000-fold greater than that found in the epididymis and the lung. Thus, *Sox30* was expressed exclusively in adult testes. However, high expression of *Sox30* in extra-gonadal tissues in other developmental stages still cannot be completely excluded.

Previous reports showed that Sox30 was expressed in normal adult testis and specifically in germ cells of mice [18], [20]. In our present study, *Sox30* expression was specifically increased coincident with the growing developmental stages until adult in the testes, and it was not only expressed in germ cells, but was also expressed in sertoli cells of mouse testes. These results indicated that Sox30 was involved in the testis development and spermatogenesis in mice. In particular, a sharp increase of Sox30 expression observed between 15d and 20d may be correlated with meiosis, which normally starts around 10d and continues thereafter in mice [32]. While the precise function of *Sox30* remains obscure and should be revealed in a future study by gene knockout animal model.

DNA methylation plays key roles in many biological processes and is particularly important for development [32]. In mice, transcriptional activation of genes is always preceded by a decrease in DNA methylation as a result of demethylation [33], [34]. It is also known that mammalian tissues are heterogeneous in their methylation state, which is hypothesized to be the driving force of the orchestrated specific gene expression in response to tissue specification [35]. Thus, the methylation and demethylation dynamics is an important mechanism in regulating gene expression and development. In our study, it was observed that dynamic changes in the expression and methylation of *Sox30* occurred in various tissues and gonads throughout mouse development. The changes in methylation were associated with opposite changes in the expression of *Sox30*, which indicated that hypermethylation might negatively contribute to regulation of Sox30 expression. Further, demethylation treatment with the reagent 5-aza-dc, a pharmacological inhibitor of DNA methylation, could restore the expression of *Sox30* in GC2, TM3 and TM4 cells. The results indicated that hypermethylation of *Sox30* indeed mediate its transcriptional silencing directly in mouse testes. In addition, the hypermethylated and hypomethylated Sox30 MSP products were both present in kidney, spleen, pancreas and intestine, which might show that both methylated and unmethylated primer-binding sites exist. This might be due to the defect of the MSP method, which is not quantitative.

Increasing evidence suggests that DNA methylation is rather “orderly” with a clear deterministic pattern throughout development. This orderly pattern is preserved over evolutionary time, and can be thought of as evolutionary “memory”, that represents a mechanism to maintain stable cellular identities [36]–[38]. On the other hand, the DNA methylation state proceeds over embryonic and postnatal development, and it is hypothesized as a driving force to direct life events, including shaping development, defining the stages of life, and determining cell fate for tissue specification [39]. Recently, new findings revealed that DNA methylation is not random, nor fixed, but is an orchestrated event during development [39]. It is spatiotemporally programmed in growing embryo, and impeding this program is found to disrupt developmental progression leading to defects [39]. Our study showed that the methylation and demethylation dynamics of *Sox30* are unveiled in various adult tissues and testes at different developmental stages. This expression and DNA methylation patterns of *Sox30* is likely to be an orchestrated event, which is essential for testis development in mice. However, it remains unknown whether or not impeding this program will disrupt developmental progression and subsequently leads to testis deficits in mice. Detailed studies will be required to answer this question.

Previous study had revealed that DNA hypermethylation is maintained until birth when germ cells resume their cycle during spermatogenesis, and the DNA methylation decreased along with successive divisions corresponding to demethylation after birth [32], [40]–[42]. In the present study, DNA methylation of Sox30 had decreased between 10d and adult testes, which may be due to the decreased expression and activity of Dnmt1 (a DNA methyltransferase) in mouse testes between 6d and the adult stage of development [43], [44]. Moreover, both de novo gene methylation and demethylation events also usually occur after completion of DNA replication during meiotic prophase [45]. These results suggested that DNA methylation of Sox30 was needed for spermatogenesis in mice. In addition, many testis-specific genes are demethylated and expressed in the testis, and are methylated in non-expressing somatic tissues [32]. This methylation and expression pattern is also suitable for Sox30.

## Conclusions

Our data demonstrated that *Sox30* expression was under the control of an epigenetic mechanism mediated by DNA methylation in testis development of mouse for the first time.
